# Chromatin position in human HepG2 cells: Although being non-random, significantly changed in daughter cells

**DOI:** 10.1016/j.jsb.2008.10.007

**Published:** 2009-02

**Authors:** Zuzana Cvačková, Martin Mašata, David Staněk, Helena Fidlerová, Ivan Raška

**Affiliations:** Institute of Cellular Biology and Pathology, First Faculty of Medicine, Charles University in Prague, and Department of Cell Biology, Institute of Physiology, Academy of Sciences of the Czech Republic, v.v.i., Albertov 4, 128 00 Prague 2, Czech Republic

**Keywords:** Live cell microscopy, Labeled chromatin region, Daughter cells, Maintenance of chromatin position, Nucleolus and nucleolus-associated chromatin

## Abstract

Mammalian chromosomes occupy chromosome territories within nuclear space the positions of which are generally accepted as non-random. However, it is still controversial whether position of chromosome territories/chromatin is maintained in daughter cells. We addressed this issue and investigated maintenance of various chromatin regions of unknown composition as well as nucleolus-associated chromatin, a significant part of which is composed of nucleolus organizer region-bearing chromosomes. The photoconvertible histone H4-Dendra2 was used to label such regions in transfected HepG2 cells, and its position was followed up to next interphase. The distribution of labeled chromatin in daughter cells exhibited a non-random character. However, its distribution in a vast majority of daughter cells extensively differed from the original ones and the labeled nucleolus-associated chromatin differently located into the vicinity of different nucleoli. Therefore, our results were not consistent with a concept of preservation chromatin position. This conclusion was supported by the finding that the numbers of nucleoli significantly differed between the two daughter cells. Our results support a view that while the transfected daughter HepG2 cells maintain some features of the parental cell chromosome organization, there is also a significant stochastic component associated with reassortment of chromosome territories/chromatin that results in their positional rearrangements.

## 1 Introduction

In the post-genomic era, when human genome had been sequenced, we possess a large amount of information about individual genes. However, the function of DNA is not entirely determined by its linear sequence and is, to a large extent, affected by the higher order organization of chromatin fibers and the three-dimensional arrangement of chromosome territotories (CTs)^2^/chromatin (e.g. Fraser and Bickmore, 2007; Misteli, 2007). It has been demonstrated that chromosomes in the interphase nucleus occupy mutually exclusive CTs (e.g. Cremer and Cremer, 2001; Lanctot et al., 2007). A more recent high-resolution in situ hybridization procedure revealed intermingling of CTs at their borders (Branco and Pombo, 2006). Regardless of their transcriptional activity, genes could be, except some specific cases (Clemson et al., 2006), found anywhere within a CT (e.g. Belmont et al., 1999), and active gene regions were even found to loop out from CTs (Volpi et al., 2000; Williams et al., 2002).

Based on a statistical analysis of large sets of mammalian cells, numerous studies have shown that the CTs are non-randomly arranged within the nuclear space (e.g. Parada and Misteli, 2002). CTs were shown to be preferentially organized radially according to their size and according to their gene density; and even chromosomal bands within a given chromosome were shown to be organized radially according to their density (Boyle et al., 2001; Kupper et al., 2007; Federico et al., 2008; e.g. Parada and Misteli, 2002; Lanctot et al., 2007). It was also reported that CTs have non-random neighborhoods as they occupied preferential positions relative to one another (Nagele et al., 1999—however, see Bolzer et al., 2005; Parada et al., 2003; e.g. Parada and Misteli, 2002). Several contacts between different chromosomal loci were reported, which may contribute to gene silencing or activation (e.g. Cavalli, 2007; Meaburn et al., 2007).

Several reports showed that the CT/chromatin order is, within nuclei, to a large extent stably maintained during interphase in cultured mammalian cells, except the early G1 phase of the cell cycle during which some increased chromatin mobility was observed (Gerlich et al., 2003; Walter et al., 2003; Thomson et al., 2004; Wiesmeijer et al., 2008). At the same time, it is to be emphasized that, during development, differentiation or upon experimental modulation of cell metabolism, not only smaller, but even extensive changes of CT/chromatin or chromatin domains nuclear position are observed (e.g. Chuang and Belmont, 2007; Kumaran et al., 2008); it should be noted, however, that some data discussed in the review by Kumaran et al. (2008) have been retracted meanwhile (Nunez et al., 2008a,b).

The non-random organization of chromosomes in mammalian nuclei raises a question: Is, or is not, the CT/chromatin position, together with its neighbourhood, preserved through mitosis in daughter cells? This question has already been addressed with divergent results obtained (Gerlich et al., 2003; Walter et al., 2003; Thomson et al., 2004; Essers et al., 2005; Berr and Schubert, 2007; e.g. Bickmore and Chubb, 2003; Gerlich and Ellenberg, 2003; Parada et al., 2003; Williams and Fisher, 2003). It should be noted that the prototype studies by Gerlich et al. (2003), Walter et al. (2003) and Essers et al. (2005) claiming either preservation of chromatin position or its significant positional changes in daughter cells, were performed on selected nuclear chromatin regions—in the form of the nuclear pole, the sector of nucleus and the half of nucleus—of unknown content and function.

In this respect, the cell nucleus contains many structures/bodies such as nucleolus, Cajal bodies and PML bodies that are linked with a particular nuclear function, and often associate with distinct chromosomal sites (Frey et al., 1999; Schul et al., 1999; e.g. Parada and Misteli, 2002). The best characterized example of nuclear bodies-chromosomal loci associations is the nucleolus (e.g. Raska et al., 2006; Boisvert et al., 2007). Beside other functions, the nucleolus is the structure in which rRNA is synthesized and ribosome biogenesis takes place. Ribosomal genes are arranged in arrays of head-to-tail tandem repeats called nucleolus organizing regions (NORs), which are in human cells located within the short arms of 5 acrocentric chromosomes 13, 14, 15, 21 and 22. Human cell nuclei typically contain one to several nucleoli. At the onset of mitosis ribosomal genes cease to transcribe and nucleoli disintegrate. rRNA synthesis resumes at the end of mitosis, ribosomal arrays from more than one NOR-bearing chromosome (NOR-chromosome) then often cluster, and respective nucleolus is reformed around them. Reformation of the nucleolus in early G1 phase thus proceeds through interactions between different CTs, and the nucleolus-associated chromatin (NAC) corresponds significantly, but not exclusively, to parts of NOR-chromosomes with ribosomal genes being rather engulfed within nucleoli (Manuelidis and Borden, 1988; Leger et al., 1994; Smirnov et al., 2006; Kalmarova et al., 2007). In the electron microscopic image, a rim of heterochromatin typically surrounds nucleoli as the result. An investigation whether the NAC position is, or is not, preserved across mitosis would then have higher relevance than that of chromatin regions of completely unknown content and function.

It should be noted that the exact molecular mechanism for the existence/maintenance of nucleoli is still to be established. The involvement of NORs and transcription of ribosomal genes represent indeed a key condition (e.g. Raska et al., 2006). However, it is not a sufficient condition due to the presence of numerous protein and RNA molecule factors affecting the nucleolar function/structure (Gonda et al., 2003; Peng and Karpen, 2007, and references there in).

In this work, we re-investigated the behavior of previously described chromatin regions of unknown composition and investigated the behavior of NAC through mitosis up to the next interphase in daughter cells. We took advantage of newly developed green to red photoconvertible protein Dendra2 (Gurskaya et al., 2006) that allowed us, via fusion protein histone H4-Dendra2, to selectively label different chromatin regions in transfected human HepG2 (HepG2^H4-Dendra2^) cells, monitor by time-lapse imaging large number of cells and apply quantitative analysis of labeled chromatin distribution in mother and daughter cells.

## 2 Materials and methods

### 2.1 H4-Dendra2 construct and HepG2 ^H4-Dendra2^ stable cell line

H4-Dendra2 was generated from full-length of human histone H4 (from pBOS H4-N-GFP expression vector, kind gift from H. Kimura), which was flanked by PCR with XhoI and EcoRI restriction sites and ligated into those sites of pDendra2-N (Evrogen).

The construct was transfected into uneuploid HepG2 cells using Effectene (Qiagen) and stable clones were selected with G418. One clone with bright Dendra2 fluorescence was chosen and expanded into the cell line. HepG2^H4-Dendra2^ stable cell line was cultured in Dulbecco’s modified Eagle’s medium with 10% fetal bovine serum, supplemented with penicillin–streptomycin, but without G418. For microscopy, cells were cultured in glass bottom Petri dishes (MatTek). Live cell imaging was performed in phenol red-free medium. It should be noted that parallelly with HepG2 cells, we tried in vain to obtain a stably transfected HeLa cell line.

Besides it, in order to evaluate nucleolar counts, HepG2, HepG2^H4-Dendra2^, HeLa (Kalmarova et al., 2008) and human primary LEP fibroblast (Sevapharma) cells were cultured under standard conditions.

### 2.2 Dendra2 photoconversion and live cell imaging

Live cell imaging was performed with Leica TCS SP5 accessorized with large size temperature incubator with CO_2_ controller (Life Imaging Services) and using 63×/1.4 NA oil immersion objective.

Non-activated Dendra2 exhibited green fluorescence visualized with 488 nm Ar laser. To label specific chromatin region, Dendra2 was activated with 405 nm diode laser line. Photoconverted Dendra2 exhibited a bright red fluorescence (visualized with 561 nm DPSS laser line), while the green fluorescence was partially bleached. It should be noted that we tried in vain to activate Dendra2 with accessible Leica TCS SP2 equipped with 2-photon excitation.

For time-lapse experiments, cells were partially synchronized by incubation with 3 mM thymidine (Sigma) for 16 h. The same experiments were also performed with non-synchronized cells and, although a lower number of cells could be analyzed, entirely compatible results on labeled chromatin were obtained (Cvačková, unpublished).

Time-lapse was mostly set to 30 min intervals and by sequential scanning of green and red fluorescence of H4-Dendra2 was visualized overall chromatin and chromatin marked by photoconversion, respectively. At low power of 488 nm laser used, such repeated scanning of green fluorescence did not lead to any observable Dendra2 activation if performed in control experiments, in which Dendra2 conversion was omitted (Cvačková, unpublished). Bright field was also captured to control the morphology of cells. Time-lapse imaging was run overnight. Prolonged time-lapse imaging until the third interphase was also performed.

More than 500 cells were evaluated in time-lapse experiments in total and a special care was paid to the deleterious phototoxic effect. In order to limit its extent, we restricted the acquisition of 3D scans in most experiments to the initial moment just after photoconversion and to the final moment at the end of the time-lapse experiment.

### 2.3 Western blot analysis

Whole-cell lysates were separated on 12% SDS–PAGE and transferred to nitrocellulose membranes (Protran). The membranes were incubated with rabbit anti-histone H4 (Abcam) or rabbit anti-Dendra (Evrogen) and then goat anti-rabbit secondary antibodies conjugated with horseradish peroxidase (Jackson) in a standard way. Horseradish peroxidase activity was detected using an ECL chemiluminescence system (Pierce) and captured with X-ray film (Foma).

### 2.4 Length of the cell cycle

Doubling time of unsynchronized cultured cells was 18.68 ± 2.24 h. To ensure that the beginning of the time-lapse experiments with partially synchronized cells was set several hours prior mitosis and the end at least 4 h after mitosis, relative ratio of cell cycle phases (G1, S and G2 together with mitosis) was in unsynchronized cells established using flow cytometry of propidium iodide labeled cells (Beckman Coulter, Cell Lab Quanta); in addition, S phase length was evaluated on the basis of the immunocytochemistry of incorporated 5-bromo-2-deoxyuridine (Sigma) (Cvačková and Mašata, unpublished).

### 2.5 Image processing and data evaluation of labeled chromatin regions of unknown composition

To quantitatively analyse the nuclear distribution (degree of compactness) of labeled chromatin (the nuclear pole, the segment of nucleus and the half of nucleus) in the mother and the daughter cell nuclei, averaged distance (pixels of 60 nm) of photoconverted signal from the gravity center of this signal was measured. In the case of identical distribution there should be similar averaged distances in the mother and the daughter cells. To compare these distances to the situation mimicking a random distribution of chromosomes in nuclei, we also calculated these average distances over whole nuclei. For clearness, the real averaged distance is given as the percentage of the random distribution distance.

All evaluations were done on thresholded (binary) images. As the choice of correct threshold is highly subjective, it was performed manually only for photoconverted signal in mother cells in which the threshold values were easy to determine (in Photoshop). For daughter cells, the threshold was calculated automatically under condition that there was the same amount of activated pixels as in mother cells (in Matlab). It should be noted that the calculated averaged distances were not sensitive to small variation in the threshold setup.

### 2.6 Fluorescent in situ hybridization of chromosomes and number of nucleoli

The immunocytochemical visualization of nucleoli via fibrillarin mapping and the in situ hybridization of all NOR-chromosomes 13, 14, 15, 21 and 22, as well as of chromosomes 6, 10 and 18, was performed as in Kalmarova et al. (2007).

To compare numbers of nucleoli in the two daughter cells, the mitotic cells were seeded, processed 4 h after seeding for the immunocytochemical visualization of nucleoli, and nucleolar counts in 100 pairs of daughter cells were evaluated. In four experiments, only 19, 20, 24 and 24% pairs exhibited the same number of nucleoli, and the values 22.0, 21.4, 22.0 and 21.8% of the corresponding random pairing model (Kalmarova et al., 2008) fitted well these counts. Furthermore, the incidence of the same number of nucleoli in the three cells (i.e. the mother cell and the two daughter cells) was in time-lapse experiments with partially synchronized cells ranging between 3% and 7%. Results of nucleolar counts in daughter cells, performed in the same way with HeLa cells and non-transfected HepG2 cells (Kalmarova et al., 2008; Smirnov and Kalmarova, unpublished), matched perfectly those observed in HepG2^H4-Dendra2^ cells. In addition, analogous experiments with human primary LEP fibroblasts exhibiting lower number of nucleoli (mean number 2.78 nucleoli versus 3.63 value observed in HepG2^H4-Dendra2^ cells) showed that more than 67% of pairs of daughter cells exhibited different nucleoli numbers.

### 2.7 Image processing and data evaluation of the nucleolus-associated chromatin

Best focus light-optical sections were further processed. First, the areas of the nucleoli and the nucleus were manually defined in RGB channels in Photoshop CS software according to bright field and H4-Dendra2 signal, respectively. Then, employing a routine written in MatLab software, each cell was divided into 15 equivalent regions, concentrically arranged around all nucleoli within the nucleus (Fig. 4). Intensity of the red fluorescence was averaged within every region and plotted against the distance of this region from the nucleoli borders. Since the signal of converted H4-Dendra2 was split from the mother cell into the two daughter cells, the daughter cell curve was normalized (same area under the curve as in the mother cell curve) to enable direct comparability between both curves.

**Fig. 1:**
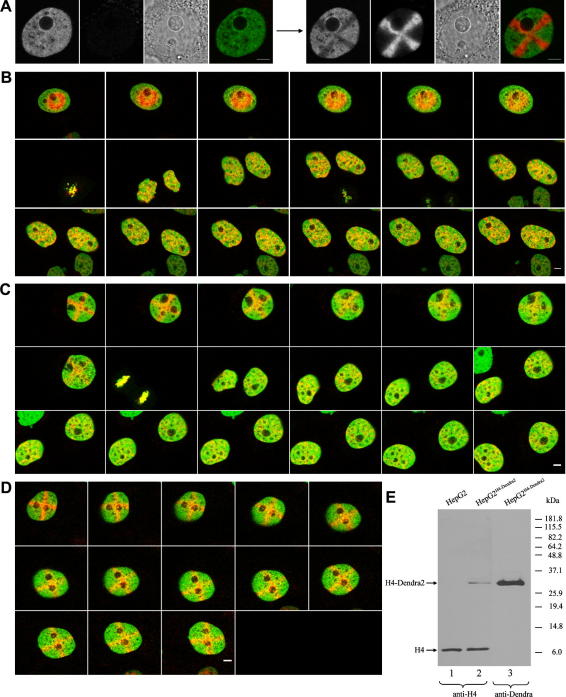
Characterization of HepG2^H4-Dendra2^ stable cell line and chromatin behavior during interphase and mitosis recorded by time-lapse imaging. (A) Non-activated Dendra2 exhibits green fluorescence. Activation results in bright red fluorescence, while the green fluorescence is partially bleached. The two channels, phase contrast and merged signals are shown. Scale bars: 5 μm. (B–D) Shortly after release from the thymidine block, regular shapes, rings (in Fig. B) and crosses (in Fig. C and D), are highlighted in chromatin by Dendra2 photoconversion and the behavior of labeled chromatin is followed across the mitosis. Individual pictures are shown in one hour intervals. Scale bars: 5 μm. In Fig. B and C, the labeling pattern exhibits some chromatin movement and/or histone exchange, but the pattern of rings and crosses remains well distinguishable from the moment of activation until the onset of mitosis. The chromatin pattern formed in later G1 differs significantly from the regular shapes labeled in the foregoing cycle, but remains again to a large extent unchanged until the end of the time-lapse imaging. In Fig. D, an example of the labeled cell, which fails to divide during a course of 12 h, is given. Note rotation of the nucleus in this Figure as well as in Fig. 4C. (E) Western blot analysis of whole cell lysates of untransfected and stably transfected HepG2 cells. Equivalent amount of proteins were loaded and incubated with anti-H4 (lines 1 and 2) or anti-Dendra antibodies (line 3). Bands corresponding to the endogenous and Dendra2-tagged histones are designated. With respect to H4-Dendra2, the relative amount of small endogenous H4 may be underestimated.

Specially with respect to the experiments with the NAC, we were aware of the deleterious effect due to the illumination cone and evaluated the extent of the 3D activation “noise”. We found it, in contrast to experiments in which the half of nucleus and the nuclear segment were labeled, of limited importance particularly with larger nucleoli (Fig. 1D). To minimize this effect, time-lapse experiments of NAC were quantitatively evaluated under condition that mother cell was mainly bearing larger nucleoli.

## 3 Results

### 3.1 Characterization of the HepG2 cells expressing photoconvertible histone H4-Dendra2

To address chromatin behavior, we first established a stable cell line carrying histone H4 tagged with the photoconvertible protein Dendra2 that was connected to the C-terminus of the histone H4 through a linker of 17 amino acid residues. This cloning strategy enabled the N-terminus of histone to be accessible for post-translation modifications (Kimura and Cook, 2001) and a linker facilitated incorporation of the fusion H4-Dendra2 protein into the nucleosome. Photoconvertible fluorescent protein Dendra2 tagged to H4 allows to label and follow selected chromatin regions (red fluorescence of converted Dendra2) and, at the same time, to monitor the whole chromatin (green fluorescence of non-converted Dendra2) in living cells (Fig. 1A). In addition, it was shown (Kimura and Cook, 2001) that the inner core histones H3 and H4 are less exchanged in the nucleosomes as compared to the outer histones H2A and H2B, and thus represent better markers for long-term observation than H2B histone used in previous studies (Gerlich et al., 2003; Walter et al., 2003; Essers et al., 2005).

HepG2 cells were chosen because generally cancer cells, that allow for the establishment of stable transfection, are less sensitive to photodamage than primary cells and therefore more suitable for long-term live cell imaging experiments (Walter et al., 2003). After Dendra2 photoconversion, 87% of cells (out of more than 500 analysed cells) passed through mitosis without morphological abnormalities or delay in the cell cycle, which indicated that the laser pulse used for the photoconversion (and image acquisitions) did not cause major defects. Moreover, photoconverted cells passed also the second mitosis during prolonged live cell imaging. However, the signal of activated Dendra2 in granddaughter cells was too weak and did not allow further evaluation (Cvačková, unpublished).

### 3.2 Chromatin position is rather stable during interphase but, although being still non-random, is significantly changed in a vast majority of daughter cells

To test chromatin behavior during interphase and mitosis, the randomly chosen chromatin regions of two regular shapes with distinct topologies, rings and crosses, were labeled within nuclei by H4-Dendra2 photoconversion and the cells were observed by time-lapse imaging (Fig. 1B–D). Cells were partially synchronized to G1/S phase boundary by one thymidine block, labeling was performed shortly after release from the block and the time-lapse was run overnight. During this time the red signal of converted Dendra2 remained detectable. The regions of labeled chromatin exhibited only minor changes from the moment of photoconversion until mitosis onset, and the labeled rings and crosses remained clearly distinguishable. During mitosis and early G1 phase, labeled regions of chromatin were rearranged. The pattern of labeled chromatin formed in later G1 phase typically largely differed from regular shapes labeled in the previous cell cycle. However, similarly to the situation encountered in the previous cell cycle, this pattern of labeled chromatin was rather stable, exhibiting just minor changes until the end of time-lapse (Fig. 1B and C).

**Fig. 2:**
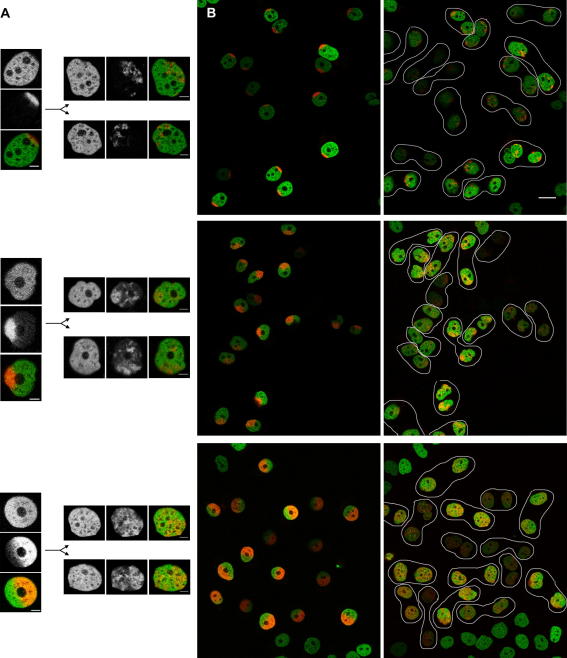
The behavior of the labeled nuclear domains of unknown composition. The behavior of the nuclear pole chromatin, the nuclear sector and the half of the nucleus is shown (consecutively) in the time-lapse experiments. Photoconversion was performed approximately 4 h after release from the thymidine block. The time-lapses corresponded to 13, 16 and 17.5 h, respectively. (A) Examples of images of the mother cell (overall chromatin, photoconverted chromatin and merged image) and the daughter cells are provided. Scale bar: 5 μm. (B) The initial and the final merged snapshots of the time-lapse are shown. The pairs of daughter cells are indicated. Note extensive changes in the distribution of label in many daughter cells. Scale bar: 20 μm.

**Fig. 3:**
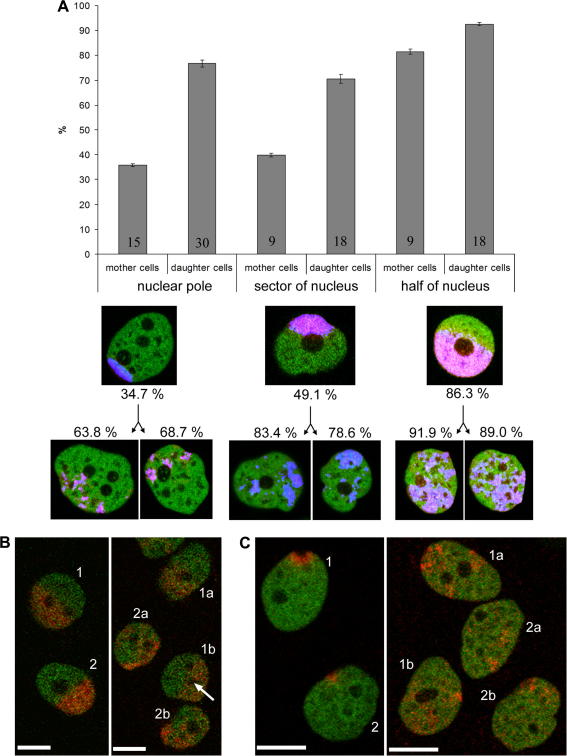
The behavior of the labeled nuclear domains of unknown composition and the quantitative evaluation of the signal distribution in mother and daughter cells. (A) Quantitation of the threshold images (the real averaged distance is given as the percentage of the random distribution distance; the number of evaluated cells is given in individual columns) together with examples of cells shown already in Fig. 2A. The data show that the distribution of signal in daughter cells is not identical with that in mother cell but is not, at the same time, randomly scattered. (B) An example of the cell in which the distribution of label in the daughter cells is similar to that seen in the mother cell (relevant cells are designated as 1, 1a and 1b). Here the position of nucleoli testifies for extensive changes of the chromatin position that took place in the daughter cells. In the mother cell 1, the two nucleoli are situated at the border of the photoconverted chromatin region. However, in the daughter cell 1b, an arrow points to the nucleolus that is entirely engulfed within the labeled chromatin region. The shown images were taken 6 h prior mitosis and 4 h after the mitosis was completed. Scale bar: 10 μm. (C) Example of two mother cells in which a tiny perinuclear chromatin region was photoactivated. In the daughter cells, the label is scattered; to improve visibility of the scattered signal, the image was contrast-stretched. Live cell imaging experiment lasted 18 h. Scale bar: 10 μm.

To further evaluate a general chromatin position during the cell cycle we expanded our study and randomly labeled, approximately 4 h after release from the block, three different regions of the nuclear chromatin as in previously published studies: the nuclear pole, the sector of nucleus and the half of nucleus (Gerlich et al., 2003; Walter et al., 2003; Essers et al., 2005). The distribution of the labeled chromatin in the daughter cells was different in a vast majority of daughter cells; it did not achieve the mother cell-like compact shape and the signal of converted H4-Dendra2 was scattered in most cells (Figs. 2, 3A and B). Cells showing a labeled pattern, that to some extent resembled the situation in mother cells, were not frequent and their occurrence in individual live cell imaging experiments ranged between 0% and 25%.

We have to emphasize that the major drawback in this kind of experiments is to be attributed to the unknown chromatin composition of the labeled region. Accordingly, within the frame of one single live cell imaging experiment, the labeled chromatin regions in different mother cells had different composition. And even if the pattern of the label seen in the daughter cells resembled to some extent to that seen in the mother cell, we can hardly say anything about the chromatin/CT order preservation within the labeled region, except if some additional markers apply such as nucleoli (Fig. 3B).

This being said, the quantitative evaluation of data with labeled regions of unknown composition showed that the distribution of signal in the daughter cells was not identical with that in the mother cell but is not, at the same time, scattered randomly as values in the daughter cells neither approached the values seen in mother cells, nor reached value 100% (Fig. 3A). In fact, whatever was the selected chromatin region, the average distribution of label in daughter cells corresponded to the distribution being roughly in a midway between identical and random distribution (Fig. 3A), i.e. the distribution was non-random. It could be also seen that the calculations are mainly sensitive for small activated regions (the nuclear pole) while there was not enough space for chromosomes to move if a large region was activated (the half of nucleus); the sector region experiments provided intermediate results. With respect to the value of significance ascribed to experiments with photoconverted chromatin regions of unknown composition, the choice of the nuclear pole region is to be considered as by far the best choice since the photoconversion affected at most several chromosomes only. The results of live cell imaging experiments with the nuclear pole region provided straightforward images of scattered labeled chromatin (Figs. 2, 3A), and necessarily argued against the preservation of chromatin position, at its large scale organization, in most cells.

An important issue also concerns the number of (fully or partially) labeled CTs within the labeled region. As already mentioned, the nuclear pole region is the best choice as only several CTs were labeled at most while numerous CTs were labeled in the sector of nucleus and the half of nucleus nuclear regions. In order to minimize the number of labeled CTs, we photoconverted the smallest possible region in the nuclear periphery affecting just a very few CTs under condition that the signal could be still evaluated in the daughter cells. We observed that the distribution of signal in many cells, but not in all daughter cells, was scattered into two or more labeled regions and thus largely differed from that seen in the mother cell (Fig. 3C).

The results obtained with labeled chromatin regions of unknown composition in HepG2^H4-Dendra2^ cells showed that the chromatin position is from the time of photoconversion to the onset of mitosis as well as from the later G1 phase until the end of live cell experiment rather stable, displaying only minor changes. Extensive changes in the distribution of the chromatin signal are observed after mitosis in most daughter cells, but the chromatin position still has non-random character.

### 3.3 Different nucleoli numbers are incompatible with the preservation of CT/chromatin position in daughter cells

The inconvenience of above mentioned experiments was completely unknown composition of labeled chromatin regions. In order to circumvent this problem, at least to some extent, we turned to the NAC as it is known that a significant part of it contains DNA sequences belonging to NOR-chromosomes. Accordingly, we first established the association of various chromosomes with nucleoli via combined mapping of relevant chromosomes by fluorescence in situ hybridization (FISH) and nucleoli by immunocytochemistry. We also evaluated the number of nucleoli in nuclei of HepG2^H4-Dendra2^ cells.

In agreement with our previous studies with human HeLa cells and primary human fibroblasts (Smirnov et al., 2006; Kalmarova et al., 2007, 2008), human NOR-chromosomes (chromosomes 13, 14, 15, 21 and 22) exhibited frequent associations with nucleoli of HepG2^H4-Dendra2^ cells, with FISH signal penetrating sometimes within nucleoli. We also performed FISH with chromosomes 6, 10 and 18 and observed some associations of these chromosomes with nucleoli, but the frequency of associations was considerably less than that with the NOR-chromosomes (Cvačková, unpublished results).

HepG2^H4-Dendra2^ cells exhibit usually one to five nucleoli. The counting of nucleoli revealed significant differences between the two daughter cells as also shown in parallel experiments with HeLa cells by Kalmarova et al. (2008) and non-transfected HepG2 cells (see Section 2). Only less than 25% of daughter HepG2^H4-Dendra2^ cell pairs (see Section 2) exhibited the same numbers of nucleoli. This finding demonstrated that nuclear positioning of at least all chromatin NOR domains were not identical in more than 75% pairs of daughter cells. As nucleoli were in nuclei usually found separated by several micrometers, our results also indicated that different sets of NOR-chromosomes differently associated with individual nucleoli in a majority daughter cells, this finding being incompatible with the chromatin position preservation in the daughter cells.

Importantly in this respect, different numbers of nucleoli were also present in a majority of daughter cell pairs of primary human fibroblasts (see Section 2). More than 67% pairs of daughter cells exhibited different numbers of nucleoli. This percentage was lower than that in transformed investigated cells but it should be noted that the average number of nucleoli within nuclei of primary cells was also lower (see Section 2).

In summary, our results with HepG2^H4-Dendra2^ cells are in agreement with the established fact that a significant part of the NAC corresponds to chromatin of NOR-chromosomes. Our results demonstrating the differences in nucleolar counts are not in harmony with the preserved CTs/chromatin position in a majority of daughter cells.

### 3.4 The NAC is dispersed in daughter cells and differently associates with different nucleoli

To verify the conclusions arising from nucleolar countings also in living cells as well as to investigate behavior of chromatin region, the significant part of which is composed of parts of NOR-chromosomes, we selectively labeled the NAC in mother cells and followed the fate of labeled chromatin up to the next interphase in the daughter cells.

**Fig. 4:**
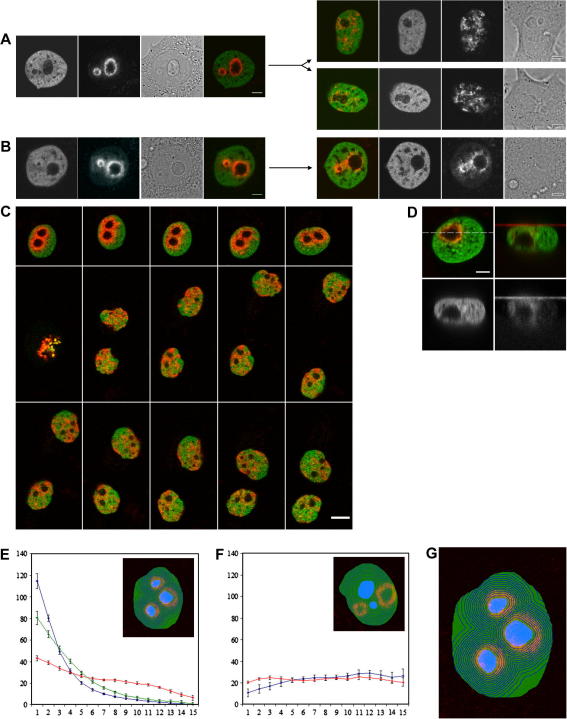
The labeling of the NAC, the behavior of the labeled NAC in live cell experiments and the quantitative evaluation of the location of the mother cell-labeled NAC with nucleoli in the daughter cells. The chromatin in the closest vicinity of nucleoli in HepG2^H4-Dendra2^ cells is photoconverted in S/early G2 phase. Labeled cells are observed by time-lapse imaging. (A) Initial snapshot taken just after conversion and the snapshot taken 14 h later are documented (the two channels, the bright field and the merged image are shown). Scale bar: 5 μm. (B) Similar as in A, but the labeled cell failed to divide during 15 h. Scale bar: 5 μm. (C) Time-lapse imaging of the mother cell-labeled NAC. Merged image snapshots in one hour intervals are shown. Note that during the time-lapse imaging with the focal plane fixed, the dynamic behavior of daughter cells resulted sometimes e.g. in disappearance and reappearance of nucleoli in some snapshots. Scale bar: 10 μm. (D) Chromatin labeling at the time of photoconversion is provided. Merged image of *xy* section (dashed line corresponds to the plane of *xz* section) and *xz* section, as well as individual channels of converted and overall chromatin in *xz* sections, are shown. Scale bar: 5 μm. (E–G) Evaluation of the distribution of the photoconverted H4-Dendra2 signal with respect to nucleoli in mother and daughter cells. Acquired images (fluorescence of non-converted and converted Dendra2; bright field pictures) are processed as described in Section 2. Outcomes are dependencies of the average pixel intensity of converted Dendra2 in the given region related to the distance of the region from nucleoli borders. Blue curves represent chromatin behavior in mother cells, red curves are related to chromatin in daughter cells. Inserts in Fig. E and F illustrate segmentation of the nucleus into equivalent regions concentrically arranged around all nucleoli within the nucleus. In Fig. E, photoconverted chromatin in both mother (*n* = 24; mean and standard deviation of the mean are given) and daughter (*n* = 33) cells exhibits tendency to be associated with nucleoli. Green curve describes behavior of chromatin in cells which fail to divide (*n* = 9). The area delimited below red and above green curves roughly represents a fraction (about 30%) of photoconverted chromatin not located in the vicinity of nucleoli in daughter cells. In Fig. F, the “randomly” (i.e. not encompassing nucleoli) chosen chromatin in mother cells (*n* = 8) exhibits no bias for the nucleolar vicinity in daughter cells (*n* = 10). In Fig. G, the detailed example of the segmentation is given.

A ring of chromatin was labeled by photoconversion of H4-Dendra2 at the closest vicinity of nucleoli that were revealed by bright field imaging (Fig. 4). The extent of labeling due to the contribution of the illumination cone was found to be of a limited importance (Fig. 4D). Cells were partially synchronized by one thymidine pulse and released from the block approximately 4 h prior to photoconversion. Thus, majority of cells were in S or G2 phase as also confirmed by immunocytochemistry of incorporated 5-bromo-2-deoxyuridine (Cvačková and Mašata, unpublished). The ring-shaped pattern of labeled chromatin remained well distinguishable until mitosis onset, although a minor chromatin movement/histone exchange could be seen. Distribution of labeled chromatin in daughter cells was seen in high-resolution images that were taken several hours after mitosis (Fig. 4A). At that time, the daughter cells were passing through later G1 or even later interphase, i.e. the period when the chromatin pattern was expected to become rather stable; indeed, the labeled pattern in daughter cells remained rather stable, exhibiting just minor changes, from later G1 until the end of time-lapse imaging. However, the pattern was dispersed and largely differed from the mother cell-labeled pattern in all monitored cells (Fig. 4A and C; video shown in Supplementary material), this being not in harmony with a concept of the chromatin position preservation.

The video (30 min snapshot intervals) showing the behavior of the labeled NAC in the mother and the daughter cells encompassed 15 h. Note that only partially synchronized cells were used and one mother cell was labeled at the onset of mitosis. This resulted in a special distribution of the signal in the respective daughter cells due to the already reorganized chromosomes in the mother cell.

Fluorescent images of converted and overall non-converted H4-Dendra2 chromatin together with the bright field snapshots were recorded several hours before, and several hours after, cell division (Fig. 4), and the position of labeled chromatin with respect to all the nucleoli in the given nucleus was quantitatively evaluated (Fig. 4E–G) as described in Section 2. To analyse data more accurately, we also took into account a contribution of chromatin movement/histone H4-Dendra2 exchange during the live cell imaging. There were a few cells that failed to divide in the course of the whole time-lapse (Fig. 4B), which were used to determine the extent of chromatin movement/histone H4-Dendra2 exchange. Although only 2D quantitative evaluation of the signal was performed, the character of curves shown in Fig. 4E was clear-cut. Juxtaposition of labeled chromatin in these cells with distribution of labeled chromatin areas in daughter cells revealed that, even though being differently dispersed, approximately 70% of labeled chromatin located to the closest vicinity of different nucleoli in the daughter cells. Similar results, with labeled chromatin being located to the vicinity of different nucleoli in the daughter cells, were obtained if the NAC of just one out of several nucleoli present in the nucleus was labeled in the mother cell (Cvačková, unpublished).

As a control, rings of similar size were labeled in “randomly” chosen chromatin region non-containing nucleoli. Acquired snapshots were subsequently processed as in the above experiments (Fig. 4F). The labeled pattern formed in daughter cells largely differed from original one (Cvačková, unpublished) but exhibited no bias for the nucleolar vicinity.

Our live cell experiments thus showed that much of the mother HepG2^H4-Dendra2^ cell-labeled NAC signal is still associated with nucleoli in the daughter cell nuclei, this reflecting implicitly the well established fact that functional NOR domains cluster in the nucleus and NOR-chromosomes are associated with nucleoli. However, the signal has a dispersed pattern and is differently associated with different daughter cell nucleoli.

## 4 Discussion

The “inheritance” of chromosome order in the mammalian daughter cells (Gerlich et al., 2003) is without any doubt a very strong claim not only within the context of biology of the cell, but also human medicine (Roix et al., 2003; e.g. Meaburn et al., 2007). Most importantly, the last publication directly relevant to the present study (Essers et al., 2005) strengthens the claim of “inheritance” of chromosome order. Accordingly, the aim of the present study was to reconcile the previously published divergent results and, with the NAC experiments, to expand the information concerning the question “Is, or is not, the CT/chromatin position, together with its neighbourhood, preserved through mitosis in daughter cells?”

Results of numerous FISH experiments led to a consensus that chromosomes are arranged in mammalian nuclei non-randomly in cell/tissue-specific manner (Parada et al., 2004; e.g. Lanctot et al., 2007). We emphasize that this consensus for the non-random arrangement is based on statistical evaluations of hundreds of cells, but does not necessarily apply to individual investigated cells. Algorithm, through which the 3D non-random organization of chromosomes is achieved, is as yet unknown.

The GFP technologies allowed, by time-lapse imaging of living cells, to test whether the nuclear arrangement of CTs/chromatin in the daughter cells is preserved with respect to that in the mother cell. In contrast to previous studies in which recombinant H2B-GFP protein was used (Gerlich et al., 2003; Walter et al., 2003; Essers et al., 2005), we used here H4 histone that is known to be less exchanged than H2B histone (Kimura and Cook, 2001) and, via photoconversion, we also avoided the problem of newly synthesized recombinant proteins used in bleach-labeled experiments performed previously. We also paid attention to the exclusion of cells just entering mitosis during photoconversion (see video in Supplementary material), this fact, in our opinion, possibly representing one reason leading to divergent results previously published in the literature

We were careful concerning the deleterious effect of phototoxicity and we rather relied on the live cell experiments during which the 3D pictures were taken just at the beginning and at the end of the time-lapse experiments, instead those in which the 3D images were acquired throughout the whole duration of the experiment. Although all the cells were necessarily affected by the light, almost 90% of monitored cells exhibited convenient biological behavior.

Whatever was the kind of labeled chromatin region, the distribution of label did not show major changes during the investigated periods of the cell cycle from the moment of photoconversion until mitosis onset as well as from the later G1 phase of the consecutive interphase until the end of the time-lapse experiment, this result being in agreement with the findings of previous studies (Gerlich et al., 2003; Thomson et al., 2004; Walter et al., 2003; Wiesmeijer et al., 2008).

Our results from numerous live cell imaging experiments with the labeled regions of unknown composition are not consistent with a concept of the preservation of CT/chromatin position in mammalian daughter cells (Gerlich et al., 2003; Essers et al., 2005). In contrast, they are in agreement with findings reported by Walter et al. (2003), according to which significant changes of chromatin position after mitosis and early G1 were established. It was already discussed that the published contradictory results could be caused by using different cell type, cells from different species or different timing of bleach-labeling of mother cells within the cell cycle (Bickmore and Chubb, 2003). We repeated in the present study the relevant experiments that were previously described in the literature and in which divergent conclusions were reached. Importantly, the re-visited divergent issue in the present study provided entirely consistent results showing that chromatin position seen in the mother cell is extensively changed in most HepG2^H4-Dendra2^ daughter cells while still exhibiting non-random character (Fig. 3A).

We emphasize that the used methodical strategy investigating the behavior of randomly selected chromatin region of unknown composition can only be used for the proof or the disproof of the identical CTs/chromatin positioning in mother and daughter cell nuclei, and for nothing between. Just one demonstration of different distribution of the labeled chromatin in mother and daughter cell nuclei is theoretically sufficient for the disproof of identical nuclear CTs/chromatin arrangement. On the contrary, any randomly chosen photoconverted chromatin region of any pattern should not be omitted from the labeling and subsequent analysis of its distribution in mother and daughter cell nuclei for the proof of identical, i.e. “inherited”, nuclear CTs/chromatin arrangement. In our experiments, some daughter cells exhibited (to some extent) similar distribution of labeled chromatin as seen in the mother cell. But this may rather reflect the lucky random labeling of chromatin region in the mother cell, the particular chromatin domains of which fulfill some of the (unknown) rules of the non-random 3D organization (like e.g. always together at the nuclear periphery). But even if so, we have hardly any control of what happened within the labeled region (regions) seen in the daughter cells (see however Fig. 3B).

The conclusion about the significantly different arrangements of CTs/chromatin of unknown composition in daughter cells was also reached with a help of immunocytochemistry that revealed different numbers of nucleoli in most investigated cells. This finding demonstrated that the 3D arrangement of NOR-chromosomes cannot be preserved in a majority of daughter HepG2^H4-Dendra2^ cells as well as HeLa cells (Kalmarova et al., 2008), non-transfected HepG2 cells and primary human LEP fibroblasts. At the same time, importantly, Kalmarova et al. (2008) reported that although the daughter HeLa cell pairs typically had different numbers of nucleoli, the associations of NOR-chromosomes 14, as well as NOR-chromosomes 15, with nucleoli in the two daughter cells were non-random. This indicated that the distribution of the NOR-chromosomes among the nucleoli is partly conserved through mitosis.

The investigation of the behavior of photoconverted NAC, the significant part of which consists of NOR-chromosomes, across mitosis is more relevant than those concerning chromatin regions of unknown content and function. In agreement with these results, our time-lapse imaging results documenting the behavior of all labeled NACs (as well as the NAC of just one out of several nucleoli present in the nucleus being labeled) in the mother cell nucleus showed that the distribution of labeled chromatin in all monitored daughter cells was dispersed and differed from that originally labeled in the respective mother cell. Even though possibly exhibiting non-random features (Kalmarova et al., 2008), the mother cell-labeled NAC differently associated with different daughter cell nucleoli. This represented also the first indirect visualization and support of combinatory variability of NOR clustering during post-mitotic *de novo* formation of nucleoli in living cells, hitherto suggested by statistical evaluation of immunocytochemical/FISH results only.

Our results concerning both various labeled chromatin regions of unknown composition and labeled NAC, are, importantly, not contradictory to the non-random 3D organization of chromosomes (Fig. 3A). They rather reflect the elementary fact that non-random does not necessarily mean identical. The accepted non-random 3D nuclear organization of chromosomes is compatible with more than one spatial arrangement of functionally specific chromatin domains (like NORs), chromosomes (like NOR-chromosomes) and functional nuclear subcompartments (like nucleoli; different numbers of nucleoli and nucleoli differently located in the nucleus).

The algorithm of non-random, cell/tissue-specific 3D nuclear arrangement of mammalian chromosomes (Parada et al., 2004; e.g. Lanctot et al., 2007) apparently allows more than one arrangement, i.e. it possesses multiple degrees of freedom. Accordingly, a possibility exists that the rare, identical CTs/chromatin arrangement might be achieved after mitosis; however, the evidence of identity requires incomparably more data than the evidence of non-identity with the methods and strategy currently used in this type of live cell imaging.

Our data on mammalian chromatin position fit the model of nuclear self-organization (e.g. Kurakin, 2007; Misteli, 2007), with chromatin being highly dynamic and its structure-function organization encompassing, importantly, also stochastic as well as “plasticity” (adaptive) features. It is an implicit model in which the sum of all properties of a chromosome determines its position (Misteli, 2007); but still, all this is a subject to stochasticity and adaptivity, and the relevant explicit molecular mechanisms remain to be established. Even a much more straightforward task—the exact determination of molecular mechanisms that stand behind the existence/maintenance of nucleoli—is not yet settled. In terms of CTs/chromatin non-random positions, it is governed via an unknown algorithm exhibiting multiple degrees of freedom, i.e. stochastic features. We are of the opinion that the cells are confronted with multiple possibilities how the nucleus is to be reassembled, with the arrangement of CTs/chromatin becoming rather stable later on during the G1 phase of the consecutive interphase.

Our results support the findings of Walter et al. (2003) and Thomson et al. (2004) who did not claim “inheritance”, but *de novo* establishment of the CTs/chromatin positioning in daughter cells during early G1 phase, such a positioning encompassing a significant stochastic component. In this respect, Kumaran and Spector (2008) showed that the cell necessitates to go through mitosis for an appropriate localization of the investigated genetic locus to the peripheral nuclear lamina and proposed that the contribution by the lamina in establishing nuclear architecture and chromatin organization occurs during the early G1 phase.

Coming back to the results on chromatin regions of unknown composition in HepG2^H4-Dendra2^ cells, chromosomes are inherited in daughter cells, but CTs/chromatin positioning is not “inherited”, although it still complies to the rules of the non-random positioning. Specifically speaking about nucleoli, nucleoli disintegrate during mitosis, but NOR-chromosomes are inherited. Functional NOR domains from several NOR-chromosomes then cluster within the nucleus and much of the originally labeled NAC is found associated with nucleoli in the daughter cells. However, NOR-chromosomes cluster in different and variable combinations, and give rise to different numbers of nucleoli in the daughter cells. Based also on our previous results with HeLa cells (Kalmarova et al., 2008), we infer that all those chromatin rearrangements in HepG2^H4-Dendra2^ comply to the non-random nuclear 3D organization of NOR-chromosomes.

Taken together, the results of the present study support the view that chromatin position is significantly rearranged in a vast majority of HepG2^H4-Dendra2^ daughter cells, while still complying to the non-random CTs/chromatin arrangement, i.e. the CTs/chromatin arrangement being partly preserved.

## Footnotes

2 *Abbreviations used:* CT, chromosome territory; FISH, fluorescence in situ hybridization; NAC, nucleolus-associated chromatin; NOR, nucleolus organizer region; NOR-chromosome, NOR-bearing chromosome.
